# Mechanical power normalisation methods to predict ICU mortality: a retrospective cohort study

**DOI:** 10.1186/s13613-025-01562-9

**Published:** 2025-09-30

**Authors:** Reza Khorasanee, Barnaby Sanderson, Emilia Tomarchio, Patrick D. Collins, Riccardo Del Signore, Sridevi Shetty, Mara Chioccola, Francesca Pugliese, Francesca Collino, Louise Rose, Lorenzo Giosa, Luigi Camporota

**Affiliations:** 1 Department of Anaesthesia, Croydon University Hospital, London, CR7 7YE UK; 2 Department of Adult Critical Care, Guy’s and St Thomas’ NHS Foundation Trust, London, SE1 7EH UK; 3 Centre for Human and Applied Physiological Sciences, School of Basic and Medical Biosciences, King’s College London, London, WC2R 2LS UK; 4 Department of Anaesthesia, Intensive Care and Emergency, City of Health and Science Hospital, C.So Bramante 88, Turin, 10126 Italy; 5 Florence Nightingale Faculty of Nursing, Midwifery and Palliative Care, King’s College London, London, SE1 8WA UK

**Keywords:** Mechanical power, Normalised mechanical power, Mechanical ventilation, Ventilatory ratio, Ventilator-induced lung injury, Ergotrauma, Acute respiratory distress syndrome

## Abstract

**Background:**

The optimal mechanical ventilation strategy to minimise ventilator-induced lung injury (VILI) remains uncertain. Mechanical power (MP) is a key VILI determinant, but whether and how MP should be normalised to individual patient characteristics is unclear. In this study, we aimed to evaluate whether the discriminatory accuracy of MP for ICU mortality in mechanically ventilated patients improves when normalised to physiologically relevant variables that reflect individual susceptibility to VILI. We also explored whether the relationship between MP, MP_ratio_, and mortality is linear or exhibits a threshold effect.

**Methods:**

In this retrospective observational study, we extracted granular electronic healthcare record data for mechanically ventilated adults in a single centre over a seven-year period. Primary exposures were MP with five normalisations: for dead space (expressed as corrected minute ventilation, ventilatory ratio, or end-tidal to arterial CO_2_ ratio); aerated lung size (compliance), and normal idealised MP (MP_ratio_). We used logistic regression to assess associations with ICU mortality. We calculated the Area Under the Receiver Operating Characteristic Curve (AUROC) to compare discriminative accuracy of individual models. Additionally, we evaluated the linearity or presence of a threshold for the relationships between MP, MP_ratio_ and ICU mortality.

**Result:**

We included 3,578 patients in our analyses. We found MP normalised to compliance (AUROC 0.71, 95% confidence interval (CI) 0.69–0.73, *p* = 0.007 (DeLong’s test)) and MP_ratio_ (AUROC 0.71, 95% CI 0.68–0.73, *p* = 0.0014) performed better than MP alone (AUROC 0.69, 95% CI 0.66–0.71) for predicting ICU mortality. Other methods of MP normalisation were no more discriminative than MP without normalisation. The relationship between MP and MP_ratio_ with ICU mortality showed a statistically significant but small departure from linearity.

**Conclusions:**

Mechanical power normalised to compliance and MP_ratio_ had better discrimination for ICU mortality than MP, although the difference was modest and absolute predictive power remained limited.

**Supplementary Information:**

The online version contains supplementary material available at 10.1186/s13613-025-01562-9.

## Background

Invasive mechanical ventilation is an important intervention in the management of patients with acute respiratory distress syndrome (ARDS), but carries the risk of ventilator-induced lung injury (VILI) [1]. The mechanisms underlying VILI include barotrauma (excessive pressure), volutrauma (excessive volume), atelectrauma (repeated opening and closing of lung units), and biotrauma (inflammatory response to ventilation) [2]. Furthermore, there is increasing recognition that the energy applied to the respiratory system during mechanical ventilation (Mechanical power -MP) is a significant determinant of VILI - a concept termed *ergotrauma* [3]. Mechanical power quantifies the energy transferred to the respiratory system per unit of time and includes key ventilatory parameters such as positive end expiratory pressure (PEEP), tidal volume, driving pressure, inspiratory flow and respiratory rate [4]. Higher MP has been associated with increased mortality in ARDS patients [5] and experimental models [6, 7] demonstrate a direct link between MP levels and histological and pathological markers of VILI [8].

One key unresolved question is whether MP should be normalised to individual patient characteristics. While MP reflects total energy delivered, its effect on the respiratory system likely varies depending on anthropometric characteristics, and amount of functional, aerated lung tissue. This is conceptually similar to the argument made for tidal volume, where its normalisation to compliance—the driving pressure—provides superior discriminative accuracy for mortality compared to indexing by predicted body weight alone [9]. Several approaches to MP normalisation have been proposed. These include adjustment for predicted body weight (PBW) [10], lung gas volumes measured by computed tomography (CT) scan [11], and compliance [12]. Some studies suggest that MP normalised to compliance or well-aerated lung tissue is more strongly associated with mortality in ARDS [11]. Another study predicting outcome of a short weaning trial found MP normalized to respiratory system compliance was enhanced by adjustment for PaCO_2_ [13]. A novel metric, the *mechanical power ratio* (MP_ratio_), represents the ratio of delivered MP to the theoretical MP required for normal minute ventilation in a healthy individual of the same age, sex, weight and height [6]. MP_ratio_ has shown early promise in predicting treatment escalation to invasive ventilation in COVID-19 patients receiving non-invasive continuous positive airway pressure (CPAP) [14]. In a small cohort of ARDS patients, MP_ratio_ outperformed MP, PaO_2_/FiO_2_ ratio, driving pressure, and alveolar dead space for mortality prediction [15]. In addition, studies of animals with healthy lungs indicate that a MP_ratio_ above 4.5 has a threshold effect above which VILI occurs [6].

Although several studies have examined mechanical power (MP) and its normalisation in small and heterogeneous populations, none has compared these parameters in a large cohort of ventilated patients spanning the full spectrum of severity—from no ARDS to severe ARDS.

In this study, we aimed to evaluate whether the discriminatory accuracy of MP, for ICU mortality in mechanically ventilated patients, improves when normalised to physiologically relevant variables that reflect individual susceptibility to VILI. We also explored whether the relationship between MP, MP_ratio_, and mortality is linear or exhibits a threshold effect.

## Methods

### Study design and setting

We conducted a retrospective cohort study using routinely collected patient data at a tertiary centre with a quaternary severe respiratory failure service in London, United Kingdom (UK).

### Study participants

We included all patients aged 18 years or older who were admitted to the ICU between 1 January 2015 and 31 December 2021 (7 years), received invasive mechanical ventilation in a mandatory mode without spontaneous effort, and had evaluable mechanical power data within 24 h of initiating mandatory ventilation. We excluded patients who were ventilated using airway pressure release ventilation (APRV), required extracorporeal membrane oxygenation (ECMO), required renal replacement therapy for pre-existing chronic kidney disease, had an ICU stay of less than 72 h, or exhibited spontaneous breathing efforts, defined as a difference of more than one breath per minute between the set and measured respiratory rates.

### Data sources

We extracted demographic characteristics, ventilator parameters and the corresponding arterial blood gas results, physiological variables, and ICU mortality from the hospital electronic records (ICIP; Philips, Netherlands) of all patients meeting our inclusion criteria and none of our exclusion criteria.

### Mechanical power calculation and normalisation

We calculated MP using the simplified formula for pressure control ventilation as described by Becher et al. [16]. Measured minute volume (V_E Meas_) was calculated as the product of respiratory rate (RR, min^− 1^) and tidal volume (V_T_, Litres). MP was calculated whenever available data were documented in the medical record. We then calculated the maximum values in the first 24-hours of mechanical ventilation. All five defined normalisation variants were calculated using maximum MP. For normalisations requiring etCO_2_ or PaCO_2_, the closest value within 2 h of the maximal recorded MP was used. If these data were unavailable, the data was considered missing and was not imputed.

We used the following formulae for MP and the five normalisation (MP_corr_, MP_VR_, MP_CO2_, MP_Cdyn_ and MP_ratio_ ) variants

1 $$MP=~0.098~ \times {P_{Peak}} \times {V_{E~Meas}}$$

MP_corr_ was defined by the substitution of corrected minute volume (V_E Corr_) for V_E Meas_ in Eq. 1. V_E Corr_ was calculated as V_E Meas_ multiplied by the ratio of PaCO_2_ to an ideal PaCO_2_ of 5.3 kPa (40mmHg) [17].

2 $$\begin{aligned} M{P_{corr}} & =~~0.098~ \times {P_{Peak}} \times {V_{E~Corr}} \\ ~~ & =~~M{P_{}} \times \frac{{PaC{O_2}}}{{5.3}}~~ \\ \end{aligned} $$

MP_VR_ was defined as the product of MP and ventilatory ratio (VR), with VR calculated as the ratio of the product of V_E Meas_ and PaCO_2_, to the product of an ideal minute volume of 0.1 L/kg/min, an ideal PaCO_2_ of 5 kPa (37.5mmHg), and PBW [18].

3 $$M{P_{VR}}=~~MP \times VR$$

Where: $VR~=~\frac{{{V_{E~Meas}} \times PaC{O_2}}}{{0.1~ \times ~PBW \times ~5}}$ and PBW was calculated as per the ARDSnet definitions [19] of:

$$ \begin{gathered} PBW_{{{\text{\male}}}} = 50~ + ~0.91 \times \left( {Height_{{cms}} - 152.4} \right)~and~ \hfill \\ PBW_{{{\text{\female}}~}} = 45.5~ + ~0.91 \times \left( {Height_{{cms}} - 152.4} \right) \hfill \\ \end{gathered} $$

MP_CO2_ was defined as MP divided by the etCO_2_/PaCO_2_ ratio.

4 $$M{P_{CO2}}=MP \times \frac{{PaC{O_2}}}{{EtC{O_2}}}$$

MP_Cdyn_ was defined as the ratio of MP and dynamic compliance of the respiratory system (C_rs_).

5 $$M{P_{Cdyn}}=\frac{{MP}}{{{C_{rs}}}}$$

MP_ratio_ was defined as the ratio of MP and a patient-specific ideal MP.

6 $$M{P_{Ratio}}=~~\frac{{MP}}{{M{P_{Ideal}}}}$$

Where: $$M{P_{Ideal}}~=~0.098~ \times {P_{Peak~Ideal}} \times {V_{E~Ideal}}$$

MP_ideal_ was derived as per the method detailed by Pozzi et al. [15]

### Statistical analysis

We fitted six univariable logistic regression models to the dependent variable of ICU mortality with MP and each of the five defined variants as predictor variables. We calculated odds ratios (OR) with 95% confidence intervals (CI) for ICU mortality considering each MP variant individually. We constructed receiver operating characteristic (ROC) curves to calculate AUROC for each model. The difference between AUROC values for each MP variant was tested against MP using DeLong’s test [20].

To explore the linearity of relationships between MP and MP_ratio_ with ICU mortality, we calculated and plotted ORs for 11 quantiles with reference to the median quantile. Additionally, we constructed logistic regression models including quadratic terms for the relevant MP variant.

Data are presented as median and interquartile ranges (IQR) for continuous data and counts and percentages for categorical data. Complete case analysis was performed with no imputation or substitution of missing data. An alpha level of 0.05 was chosen as the threshold for statistical significance. All statistical analyses were performed using R version 4.4 [21].

### Ethical Considerations

This study was approved by the Institution (No: 14632) with the need for informed consent waived due to the use of anonymised and routinely collected data.

## Results

**Fig. 1:**
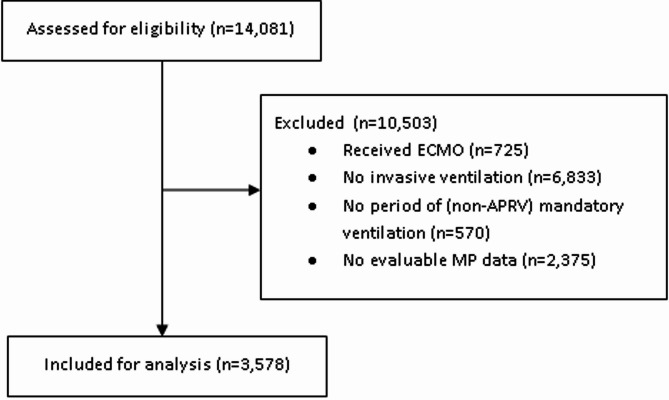
*Patient inclusion CONSORT flow diagram*

### Baseline characteristics

We included 3,578 patients in our analysis (See Fig. 1 for CONSORT diagram). Of these, 2,306 (64%) were male; median age was 60 (IQR 47, 71) years; median APACHE II score of 15 (IQR 12, 20) (Table 1). Utilising the worst PaO_2_/FiO_2_ ratio recorded in the first 24 h of invasive mechanical ventilation, we identified 763 (21.3%) patients with a PF ratio ≥ 40 kPa (≥ 300mmHg) (no ARDS); 1035 (28.9%) 26.6 kPa to 39.9 kPa (200 to 299mmHg) (mild ARDS), 1295 (36.2%) 13.3 kPa to 26.5 kPa (100mmHg to 199mmHg) (moderate ARDS), and 485 (13.6%) < 13.3kPa (< 100mmHg) (severe ARDS).

**Table 1:** *Baseline patient characteristics*

Patient Characteristic	*N* = 3,578
Age, years	60 (47–71)
Male	2,306 (64)
Height, cm	170 (165, 175)
Weight, kg	75 (65, 86)
Predicted body weight, kg	66 (57, 71)
APACHE II score	15 (12, 20)
Surgical admission	1,217 (34)
Elective admission	935 (26)
Infection related admission	762 (21)
Admission Diagnosis by Organ System:	
Cardiovascular	1052 (29)
Respiratory	1023 (29)
Gastrointestinal	538 (15)
Neurological (including eyes)	328 (9.2)
Poisoning	184 (5.1)
Genito-urinary	171 (4.8)
Endocrine, Metabolic, Thermoregulation and Poisoning	161 (4.5)
Musculoskeletal	44 (1.2)
Haematological/Immunological	31 (0.9)
Dermatological	22 (0.6)
Psychiatric	13 (0.4)
Trauma	11 (0.3)
Comorbidities:	
Cardiovascular disease	971 (27)
Pulmonary disease	772 (22)
Renal disease	448 (13)
Liver disease	181 (5.1)
Diabetes	718 (20)
Cancer	243 (6.8)
Volume control mode	232 (6)
Pressure control mode	3,346 (94)
Tidal volume, ml	537 (458, 625)
Tidal volume PBW, ml/kg	8.4 (7.1, 9.9)
Minute volume, L/min	8.9 (7.2, 10.9)
PEEP, cmH_2_O	6 (5, 8)
Peak inspiratory pressure, cmH_2_O	22 (18, 26)
Driving pressure, cmH_2_O	14 (11.5, 17.0)
Dynamic compliance, ml/cmH_2_O	38 (30, 49)
Minute volume corrected, L/min	8.5 (6.7, 11.2)
Ventilatory ratio	1.33 (1.06, 1.75)
End-tidal: arterial pCO_2_ ratio	0.88 (0.76, 1.02)

*Median (IQR) for continuous variables*,* n (%) for categorical variables. For missing data counts see Supplementary materials.*

The median maximum MP in the first 24 h of ventilation was 19 J/min (IQR 14, 26). Median values for MP normalisations are presented in Table 2.

### Outcomes

ICU mortality was 485/3578 (14%). As presented in Table 2, the AUROC values for MP variants ranged from 0.67 to 0.71. MP_Cdyn_ and MP_ratio_ demonstrated the most accurate prediction of ICU mortality. As assessed using the DeLong’s test, AUROCs for MP_corr_, MP_VR_, and MP_CO2_ were not different to MP.

**Table 2:** *Comparison of mechanical power variants*

MP Variant	Median (IQR)	OR (95% CI)	AUROC (95% CI)	*p*-value
MP	19 J/min (14, 26)	1.03 (1.02, 1.05)	0.69 (0.66,0.71)	N/A
MP_corr_	19 J/min (13, 28)	1.03 (1.03, 1.04)	0.68 (0.65, 0.70)	0.6
MP_VR_	25 J/min (15, 44)	1.01 (1.01, 1.01)	0.67 (0.65, 0.70)	0.5
MP_CO2_	22 J/min (15, 33)	1.02 (1.01, 1.02)	0.70 (0.68, 0.73)	0.4
MP_Cdyn_	0.53 J.cmH_2_O /min.ml (0.34, 0.86)	1.21 (1.11, 1.31)	0.71 (0.69, 0.73)	0.007
MP_ratio_	5.17 (3.80, 7.05)	1.23 (1.19, 1.27)	0.71 (0.68, 0.73)	0.0014

*MP variants*,* median and Meandiand interquartile ranges for each MP variant*,* odds ratios for mortality from the univariable logistic regression model and discriminatory accuracy of models as assessed by calculation of the AUROC. P-values for each MP variant using DeLong’s test was performed with reference to MP. For missing data counts see Supplementary Material.*

### Quantile analysis

**Fig. 2:**
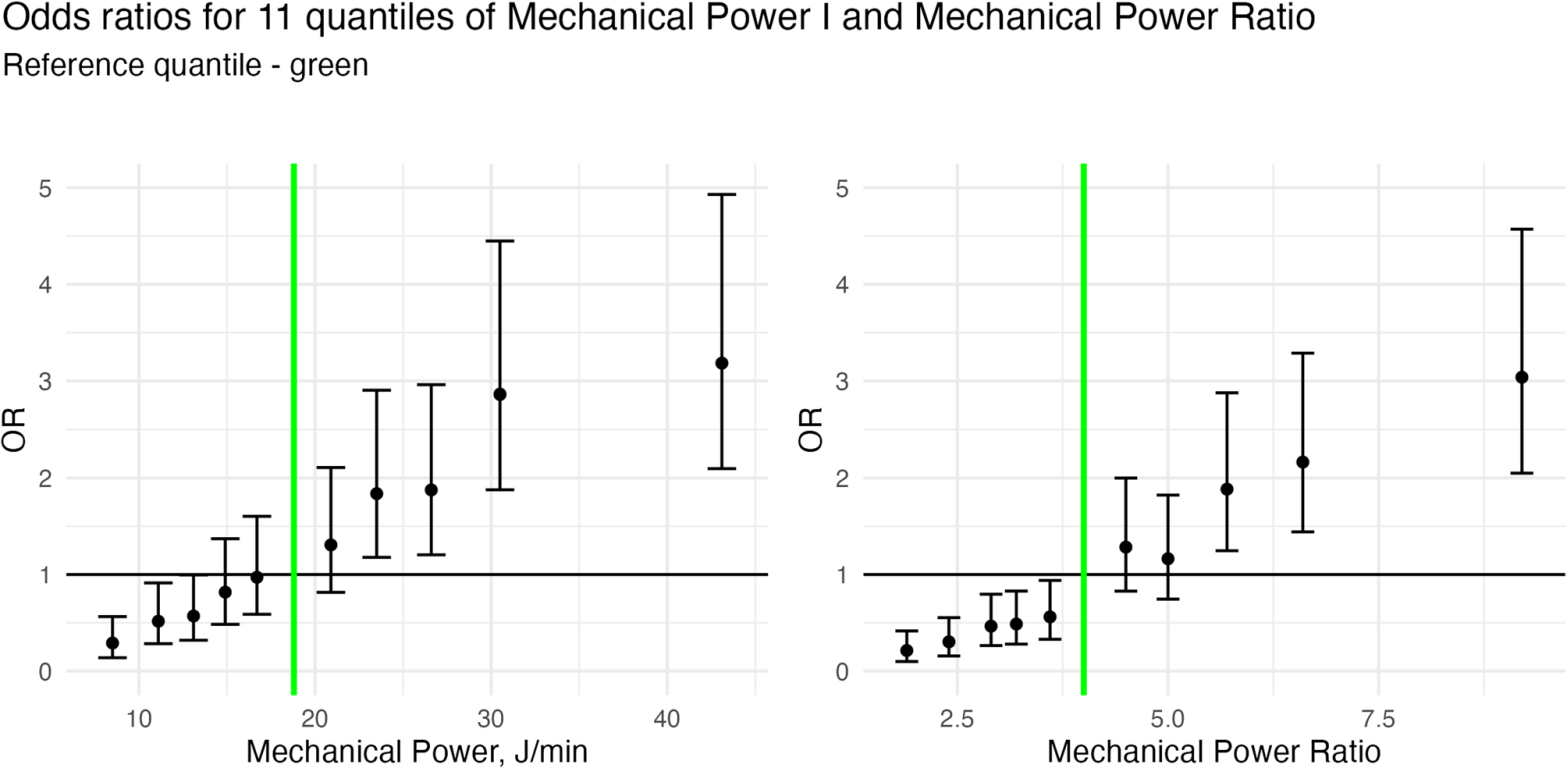
a/b *Odds Ratios for 11 Quantiles of Mechanical Power and Mechanical Power Ratio. Odds ratios for mortality plotted with 95% CIs for 11 quantiles of MP (****a****) and MP*_*ratio*_ *(****b****). Odds ratios are relative to the median quantile (green line)*

The odds ratio of ICU mortality for 11 quantiles of MP compared with the median reference quantile are plotted in Fig. 2. The inclusion of quadratic terms to logistic regression models of MP and MP_ratio_ indicated statistically significant departures from linearity for both variables. However, the quadratic coefficient was small in absolute value with minimal influence over the range of MP observed. See Supplementary Material for further details.

## Discussion

In this large cohort of mechanically ventilated ICU patients, we found that MP_ratio_ and MP normalised to compliance had a modest but statistically significant improvement in the discriminatory accuracy for ICU mortality compared to conventional MP. While the absolute increase in AUROC was limited, both measures outperformed MP normalised to dead space surrogates, which offered no additional discriminative value compared to conventional MP. Our findings are consistent with previous studies in smaller cohorts of ARDS patients that found MP normalised to compliance had a greater discriminatory accuracy than conventional MP [11, 12]. Similarly, MP_ratio_ outperformed MP normalised for calculated dead space fraction in predicting mortality in ARDS patients [15].

We selected MP normalisation variants to adjust MP based on individual patient susceptibility to VILI. These included ventilated lung size (i.e., compliance), dead space, and ventilatory efficiency. MP_ratio_ is an alternative approach, normalising MP to an ideal MP to achieve normal PaCO_2_ in healthy individuals, using idealised values of respiratory mechanics and patient-specific anthropometrics.

MP_corr_ utilises V_E corrected_ instead of V_E Measured_ as a surrogate of dead space that has been independently associated with mortality in ARDS patients [22]. MP_VR_ is conventional MP multiplied by the ventilatory ratio. First described in 2009, VR is a unitless ratio of V_E Measured_ and PaCO_2_ to predicted V_E_ (0.1 L/kg PBW) and an ideal PaCO_2_ of 5 kPa (37.5mmHg) [18]. An increase in VR may be due to increased CO_2_ production or to reduced ventilatory efficiency (i.e., increased dead space ventilation). MP_CO2_ is conventional MP divided by the ratio of end-tidal to arterial CO_2_. This ratio is used to assess gas exchange, and particularly the alveolar dead space, although venous admixture (shunt) will also decrease the value. Lung perfusion will influence this ratio although cardiac output data was not included in our data set. A low end-tidal to arterial CO_2_ ratio is strongly associated with dead space fraction, as assessed by volumetric capnography, and mortality in ARDS patients [23]. MP_Cdyn_ is conventional MP divided by the compliance which reflects the functional lung size which may be greatly reduced in ARDS patients – it has been shown to correlate with the dimensions of the baby lung [24].

Normalising MP for PBW or Crs (MP_ratio_, MP_Cdyn_) outperformed methods estimating dead space ventilation (MP_corr_, MP_VR_, MP_CO2_). This may be because MP is an extensive variable, whose absolute value depends on system size [25]. Normalising MP to lung volume (Crs) or PBW (as used in MP_ideal_) leads to an intensive variable, offering a more reliable indicator. EtCO₂ and PaCO₂ can fluctuate significantly, and using daily peak MP variant values in a heterogeneous cohort may affect their representativeness of underlying physiology.

### Threshold effects and MP relationship with mortality

Defining a threshold MP value for increased lung injury remains a key objective in ventilation research. Serpa Neto et al. found that in an ARDS cohort, MP > 17 J/min was associated with a consistent increase in mortality [5]. Manrique et al. found that the risk of ICU mortality increased by 0.1% for each hour with MP > 18 J/min [26]. In a porcine model, D’Albo et al. identified a MP_ratio_ threshold of 4.5 for increased risk of VILI [6]. In our cohort we found that there was a near-linear relationship of and ICU mortality without a significant threshold effect despite the wide range of severity in our population spanning from no ARDS to severe ARDS. This finding may suggest that, in a heterogeneous cohort of patients where the primary cause of organ failure is not necessarily the lung, using MP as a predictor of mortality may not be the variable with the highest sensitivity and specificity. Alternatively, the lack of a clear threshold value in our study may indicate the need for more robust and refined MP normalisation to better capture individual risk profiles.

Previous research suggests that MP normalisation variants may differ by patient subgroup. Zhang et al. reported that MP normalized to PBW was only significantly associated with mortality in moderate-to-severe ARDS [10]. We did not find a similar interaction between MP and PaO_2_/FiO_2_ ratio (see supplementary material) which may, in part, reflect the heterogeneity of our study population. Although PaO_2_/FiO_2_ ratio is commonly used to enrich ARDS trial design and select patients for interventions which reduce VILI risk (prone positioning, ECMO), there is increasing recognition that PaO_2_/FiO_2_ ratio alone does not necessarily correlate with the susceptibility to VILI. Therefore it may not be the ideal parameter to base respiratory support strategies on [27].

Despite incremental improvement in the discriminative ability of MP for ICU mortality, the absolute predictive performance of these models remains limited and should not be considered a replacement for established risk stratification scores. Nevertheless, our analysis of a range of MP normalisation variants offers insight into their potential physiological relevance, even if not immediately applicable for prognostic use. As MP is a variable determined not only by the pathophysiologic state but by clinical decisions in relation to ventilation strategy, any signal for an association with mortality is worth exploring. The small differences seen in this study between MP and two normalisation variants may suggest that calculating MP alone already captures the relevant signal without need for further adjustment. Alternatively, it may reflect the heterogenous nature of our cohort and the limitations of ICU mortality as an outcome measure.

### Limitations

Strengths of this study include the large, diverse cohort of invasively ventilated ICU patients.

Although the heterogeneity of our cohort supports the generalisability of our findings to a wider mechanically ventilated population, it may have masked associations within specific subgroups. Conducted at a single multi-ICU centre with a focus on respiratory failure, the study’s external validity may be limited. We advocate incorporating MP normalisation variants into prospective, phenotype-stratified studies and validating our findings in larger or open datasets to clarify the metrics’ utility and limitations.

The findings may not apply to patients ventilated using volume control, as 94% received pressure-controlled ventilation. We also did not differentiate between resistive and static elastic components of MP and VILI, but used total MP [28]. Maximal MP in the first 24 h was used to capture the most injurious early exposure and allow direct comparison of MP and its normalisations at a common time point. Future studies should examine cumulative exposure, MP trajectories, and time above threshold.

ICU mortality, though important, is an indirect marker of lung injury, influenced by extra-pulmonary failure, comorbidities, and treatment complications. Given the established link between ventilation strategy, VILI, and mortality, prospective work should assess MP normalisation against direct VILI measures such as biomarkers or imaging.

## Conclusions

In this large cohort of invasively ventilated patients, normalising mechanical power to compliance (MP_Cdyn_) and using MP_ratio_ yielded modest improvements in ICU mortality prediction compared with conventional MP. Other normalisation approaches conferred no additional discriminatory value. Although the small AUROC gains indicate only incremental improvements, none of the normalisation variants demonstrated strong predictive ability and should not be used for mortality risk stratification. Both MP and MPratio exhibited near-linear associations with mortality, without evidence of a threshold effect.

## Supplementary Information

Below is the link to the electronic supplementary material.

Supplementary Material 1.

## Data Availability

Data cannot be shared publicly due to institutional ethics, privacy, and confidentiality regulations.

## Abbreviations

APACHE II: Acute Physiology and Chronic Health Evaluation II
APRV: Airway Pressure Release Ventilation
ARDS: Acute Respiratory Distress Syndrome
AUROC: Area Under the Receiver Operating Characteristics Curve
CI: Confidence Interval
Crs: Compliance of the respiratory system
CT: Computed Tomography
ECMO: Extracorporeal Membrane oxygenation
etCO_2_: End-tidal Carbon Dioxide
ICIP: Intellivue Clinical Information Portfolio
ICNARC: Intensive Care National Audit and Research Centre
ICU: Intensive Care Unit
IQR: Interquartile Range
MP: Mechanical Power
MP_corr_: Mechanical Power substituting VE_corr_ for VE_Meas_
MP_VR_: Mechanical Power multiplied by the ventilatory ratio
MP_CO2_: Mechanical Power divided by the etCO2/PaCO2 ratio
MP_Cdyn_: Mechanical Power divided by the dynamic compliance
MP_ratio_: Mechanical Power divided by MP_ideal_
PaCO_2_: Partial pressure of arterial carbon dioxide
PaO_2_: Partial pressure of arterial oxygen
PaO_2_/FiO_2_: Partial pressure of arterial oxygen to inspired fraction of oxygen ratio
PBW: Predicted Body Weight
PCV: Pressure Control Ventilation
PEEP: Positive End-Expiratory Pressure
Ppeak: Peak airway Pressure
P-V: ressure-Volume
ROC: Receiver Operating Characteristics
RRT: Renal Replacement Therapy
UK: United Kingdom
VCV: Volume Control Ventilation
VE: Minute Ventilation
VE_corr_: Corrected minute ventilation
VE_Meas_: Actual measured minute ventilation
VILI: Ventilator-Induced Lung Injury
VR: Ventilatory Ratio
