# Supplementary material for: Mechanical power normalisation methods to predict ICU mortality: a retrospective cohort study
Source: Ann Intensive Care. 2025 Sep 30;15:149. doi: 10.1186/s13613-025-01562-9 (PMC12480304; doi:10.1186/s13613-025-01562-9)
Supplement: Supplementary file 1 — Supplementary Material 1. [file 13613_2025_1562_MOESM1_ESM.docx]

Supplemental Information

Mechanical Power Normalisation Methods to Predict ICU Mortality: A Retrospective Cohort Study

# Introduction

This document describes some additional information referenced in the journal article:

Mechanical Power Normalisation Methods to Predict ICU Mortality: A Retrospective Cohort Study

# Data

### Patient characteristics

Patient characteristics indicating counts of missing data are tabulated in [Table 1](#tbl-pt_chars)

| Table 1: Patient characteristics indicating counts of missing data, Median (IQR)   \| **Characteristic** \| **N = 3,578***^1^* \| \| --- \| --- \| \| Age, years \| 60 (47, 71) \| \| Female \| 1,272 (36%) \| \| Height, cms \| 170 (165, 175) \| \| Weight, kgs \| 75 (65, 86) \| \| Ideal body weight, kgs \| 66 (57, 71) \| \| Apache II \| 15 (12, 20) \| \| - Missing data \| 16 \| \| Surgical admission \| 1,217 (34%) \| \| Diagnosis Organ System \|  \| \| Cardiovascular \| 1,052 (29%) \| \| Respiratory \| 1,023 (29%) \| \| Gastrointestinal \| 538 (15%) \| \| Neurological (including eyes) \| 328 (9.2%) \| \| Poisoning \| 184 (5.1%) \| \| Genito-urinary \| 171 (4.8%) \| \| Endocrine, Metabolic, Thermoregulation and Poisoning \| 161 (4.5%) \| \| Musculoskeletal \| 44 (1.2%) \| \| Haematological/Immunological \| 31 (0.9%) \| \| Dermatological \| 22 (0.6%) \| \| Psychiatric \| 13 (0.4%) \| \| Trauma \| 11 (0.3%) \| \| Infection \| 762 (21%) \| \| - Missing data \| 3 \| \| Elective admission \| 935 (26%) \| \| **Co-Morbidity** \|  \| \| Cardiovascular disease \| 971 (27%) \| \| Pulmonary disease \| 772 (22%) \| \| Renal Disease \| 448 (13%) \| \| Liver disease \| 181 (5.1%) \| \| Diabetes \| 718 (20%) \| \| Cancer \| 243 (6.8%) \| \| *^1^Median (Q1, Q3); n (%)* \| \| |
| --- | --- | --- | --- | --- | --- | --- | --- | --- | --- | --- | --- | --- | --- | --- | --- | --- | --- | --- | --- | --- | --- | --- | --- | --- | --- | --- | --- | --- | --- | --- | --- | --- | --- | --- | --- | --- | --- | --- | --- | --- | --- | --- | --- | --- | --- | --- | --- | --- | --- | --- | --- | --- | --- | --- | --- | --- | --- | --- | --- | --- | --- | --- | --- | --- | --- | --- |

### Ventilation characteristics

Various parameters were extracted/calculated from observed data. Data including counts of missing data is tabulated in [Table 2](#tbl-pt_ventparams)

Due to the mismatch in charted times of ventilator observations and blood gas measurements, blood gas measurements were filled backwards within a 2 hour time interval. i.e. blood gas measurements would be considered with sets of ventilator obs within the two hours preceding them, but only if no prior (closer) blood gas measurement were made.

Six mechanical power based parameters, $MP$, $MP_{Corr}$, $MP_{VR}$, $MP_{CO_{2}}$, $MP_{Cdyn}$ and mechanical power ratio ($MP_{Ratio}$), were calculated whenever possible in the first 24 hours after a patient first receives ventilation via a “MANDATORY MODE”.

Only “MANDATORY” ventilation was considered, excluding APRV modes,

Also:

Parameters are reported from the time point where the maximum mechanical power, $MP$, was observed on the first study day where mandatory ventilation was observed.

| Table 2: Ventilation Parameters indicating counts of missing data, Median (IQR)   \| **Characteristic** \| **N = 3,578** \| \| --- \| --- \| \| Volume Contolled Mode \| 232 (6.5%) \| \| Pressure Controlled Mode \| 3,346 (94%) \| \| Tidal Volume, mls \| 537 (458, 625) \| \| Tidal Volume per kg, mls/kg \| 8.36 (7.14, 9.86) \| \| Delta Pressure, cmH2O \| 14.0 (11.5, 17.0) \| \| Compliance, mls/cmH2O \| 38 (30, 49) \| \| PEEP, cmH2O \| 6.00 (5.00, 8.00) \| \| - Missing data \| 17 \| \| Peak Airway Pressure, cmH2O \| 22.0 (18.0, 26.0) \| \| Minute Volume, L/min \| 8.91 (7.20, 10.91) \| \| Minute Volume Corrected, L/min \| 8.0 (6.3, 10.5) \| \| - Missing data \| 844 \| \| Ventilatory Ratio \| 1.33 (1.06, 1.75) \| \| - Missing data \| 844 \| \| Max MP \| 19 (14, 26) \| \| Max MP_Corr_ \| 18 (12, 26) \| \| - Missing data \| 69 \| \| Max MP_VR_ \| 25 (15, 44) \| \| - Missing data \| 69 \| \| Max MP_CO2_ \| 22 (15, 33) \| \| - Missing data \| 95 \| \| Max MP_Cdyn_ \| 0.53 (0.34, 0.86) \| \| Max MP_Ratio_ \| 5.17 (3.80, 7.05) \| \| End Tidal:Arterial PCO2 Ratio \| 0.88 (0.76, 1.02) \| \| - Missing data \| 897 \| \| **Maximum ARDS (PF) category** \|  \| \| Mild \| 1,035 (37%) \| \| Moderate \| 1,295 (46%) \| \| Severe \| 485 (17%) \| \| - Missing data \| 763 \| |
| --- | --- | --- | --- | --- | --- | --- | --- | --- | --- | --- | --- | --- | --- | --- | --- | --- | --- | --- | --- | --- | --- | --- | --- | --- | --- | --- | --- | --- | --- | --- | --- | --- | --- | --- | --- | --- | --- | --- | --- | --- | --- | --- | --- | --- | --- | --- | --- | --- | --- | --- | --- | --- | --- | --- | --- | --- | --- | --- | --- | --- | --- | --- |

## Non-linear relationship

To explore the linearity of the relationships between mechanical power (MP) and mechanical power ratio (MP_ratio_) on ICU mortality, we we constructed logistic regression models including quadratic terms for both MP and MP_Ratio_.

### Mechanical Power

A univariable model logistic regression model for MP vs ICU mortality can be described with the following formula:

$$logit\left( P\left( Y_{i}=Died \right) \right)=\beta_{0}+\beta_{1}MP_{i}$$

where $MP_{i}$ indicates the mechanical power observed for subject *i*, and outcome $Y_{i}=0$ if they survived ICU and $Y_{i}=1$ if they did not.

The R output for the logistic regression defined above was:

Call:
glm(formula = icu_mortality ~ max_mechanical_power_i, family = binomial(link = "logit"),
 data = patient_list)

Coefficients:
 Estimate Std. Error z value Pr(>|z|)
(Intercept) -2.969513 0.116372 -25.52 <2e-16 ***
max_mechanical_power_i 0.049677 0.004393 11.31 <2e-16 ***
---
Signif. codes: 0 '***' 0.001 '**' 0.01 '*' 0.05 '.' 0.1 ' ' 1

(Dispersion parameter for binomial family taken to be 1)

 Null deviance: 2839.5 on 3577 degrees of freedom
Residual deviance: 2704.8 on 3576 degrees of freedom
AIC: 2708.8

Number of Fisher Scoring iterations: 4

There is strong evidence (p<0.0001) that mechanical power is associated with ICU mortality. As the maximum mechanical power observed increases the odds of dying in ICU also increase, with an odds ratio of 1.05 (95%CI: 1.04, 1.06).

To examine whether this effect was non-linear, a similar logistic regression model was fitted which included a quadratic term for mechanical power:

$$logit\left( P\left( Y_{i}=Died \right) \right)=\beta_{0}+\beta_{1}MP_{i}+\beta_{2}\left( MP_{i} \right)^{2}$$

Call:
glm(formula = icu_mortality ~ max_mechanical_power_i + I(max_mechanical_power_i^2),
 family = binomial(link = "logit"), data = patient_list)

Coefficients:
 Estimate Std. Error z value Pr(>|z|)
(Intercept) -3.9036071 0.2127680 -18.347 < 2e-16 ***
max_mechanical_power_i 0.1164683 0.0134666 8.649 < 2e-16 ***
I(max_mechanical_power_i^2) -0.0009550 0.0001912 -4.994 5.92e-07 ***
---
Signif. codes: 0 '***' 0.001 '**' 0.01 '*' 0.05 '.' 0.1 ' ' 1

(Dispersion parameter for binomial family taken to be 1)

 Null deviance: 2839.5 on 3577 degrees of freedom
Residual deviance: 2666.6 on 3575 degrees of freedom
AIC: 2672.6

Number of Fisher Scoring iterations: 5

There was strong evidence that the coefficient for squared mechanical power, $\beta_{2}$, is not 0. Predicted probabilities across a range of mechanical power values were plotted:


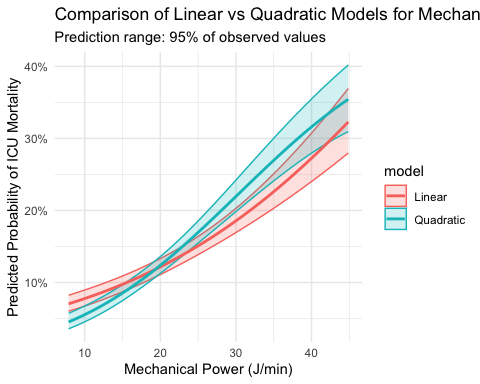


###

### Mechanical Power Ratio

A univariable model logistic regression model for MP_Ratio_ vs ICU mortality can be described with the following formula:

$$logit\left( P\left( Y_{i}=Died \right) \right)=\beta_{0}+\beta_{1}MP_{Ratio i}$$

where $MP_{Ratio i}$ indicates the mechanical power ratio observed for subject *i*.

The R output for the logistic regression defined above was:

Call:
glm(formula = icu_mortality ~ max_mechanical_power_ratio, family = binomial(link = "logit"),
 data = patient_list)

Coefficients:
 Estimate Std. Error z value Pr(>|z|)
(Intercept) -3.15488 0.11945 -26.41 <2e-16 ***
max_mechanical_power_ratio 0.20729 0.01609 12.88 <2e-16 ***
---
Signif. codes: 0 '***' 0.001 '**' 0.01 '*' 0.05 '.' 0.1 ' ' 1

(Dispersion parameter for binomial family taken to be 1)

 Null deviance: 2839.5 on 3577 degrees of freedom
Residual deviance: 2664.1 on 3576 degrees of freedom
AIC: 2668.1

Number of Fisher Scoring iterations: 5

There was strong evidence (p<0.0001) that MP_Ratio_ was associated with ICU mortality. As the observed MP_Ratio_ increasd the odds of dying in ICU also increased, with an estimated odds ratio of 1.23 (95%CI: 1.19, 1.27).

To examine whether this effect was non-linear, a similar logistic regression model was fitted which included a quadratic term for mechanical power ratio:

$$logit\left( P\left( Y_{i}=Died \right) \right)=\beta_{0}+\beta_{1}MP_{Ratio i}+\beta_{2}\left( MP_{Ratio i} \right)^{2}$$

Call:
glm(formula = icu_mortality ~ max_mechanical_power_ratio + I(max_mechanical_power_ratio^2),
 family = binomial(link = "logit"), data = patient_list)

Coefficients:
 Estimate Std. Error z value Pr(>|z|)
(Intercept) -4.239244 0.229416 -18.478 < 2e-16 ***
max_mechanical_power_ratio 0.492366 0.052978 9.294 < 2e-16 ***
I(max_mechanical_power_ratio^2) -0.015285 0.002748 -5.561 2.68e-08 ***
---
Signif. codes: 0 '***' 0.001 '**' 0.01 '*' 0.05 '.' 0.1 ' ' 1

(Dispersion parameter for binomial family taken to be 1)

 Null deviance: 2839.5 on 3577 degrees of freedom
Residual deviance: 2624.2 on 3575 degrees of freedom
AIC: 2630.2

Number of Fisher Scoring iterations: 5

There was strong evidence that the coefficient for squared mechanical power ratio, $\beta_{2}$, was not 0. Predicted probabilities across a range of mechanical power ratio values were plotted:


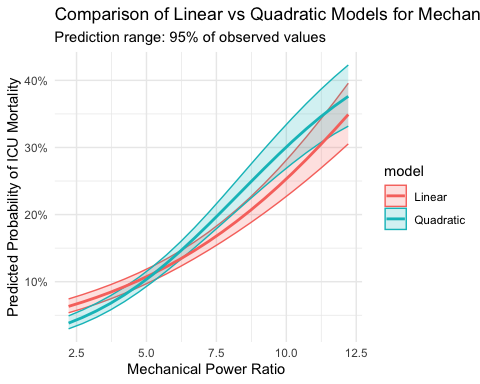


## PF ratio categories vs Mechanical Power


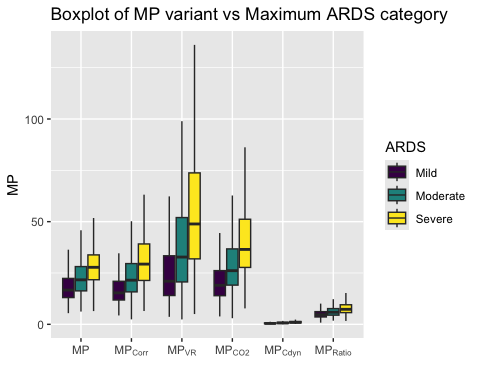


To examine the interaction of ARDS category (by P/F ratio) with mechanical power, logistic regression models were fitted with and without interaction terms between ARDS categories and mechanical power. The R summary output listed below, details interaction coefficients indicating no evidence that they differed from zero.

Call:
glm(formula = icu_mortality ~ max_mechanical_power_i * max_ards_cat,
 family = binomial(link = "logit"), data = mutate(patient_list,
 max_ards_cat = as.character(max_ards_cat)))

Coefficients:
 Estimate Std. Error z value
(Intercept) -2.800447 0.243829 -11.485
max_mechanical_power_i 0.021502 0.010880 1.976
max_ards_catModerate 0.526394 0.305885 1.721
max_ards_catSevere 0.953874 0.379124 2.516
max_mechanical_power_i:max_ards_catModerate 0.007715 0.012834 0.601
max_mechanical_power_i:max_ards_catSevere 0.011965 0.014138 0.846
 Pr(>|z|)
(Intercept) <2e-16 ***
max_mechanical_power_i 0.0481 *
max_ards_catModerate 0.0853 .
max_ards_catSevere 0.0119 *
max_mechanical_power_i:max_ards_catModerate 0.5477
max_mechanical_power_i:max_ards_catSevere 0.3974
---
Signif. codes: 0 '***' 0.001 '**' 0.01 '*' 0.05 '.' 0.1 ' ' 1

(Dispersion parameter for binomial family taken to be 1)

 Null deviance: 2490.5 on 2814 degrees of freedom
Residual deviance: 2342.1 on 2809 degrees of freedom
 (763 observations deleted due to missingness)
AIC: 2354.1

Number of Fisher Scoring iterations: 5
